# Development and Usability Study of an Open-Access Interviewer-Administered Automated 24-h Dietary Recall Tool in Argentina: MAR24

**DOI:** 10.3389/fnut.2021.642387

**Published:** 2021-08-05

**Authors:** Ismael A. Contreras-Guillén, Sara Leeson, Rocio V. Gili, Belén Carlino, Daniel Xutuc, Marcia Cristina Teixeira Martins, María E. Zapata, Gina Segovia-Siapco, Joan Sabaté, Fabio J. Pacheco, Sandaly O. S. Pacheco

**Affiliations:** ^1^School of Medicine and Health Sciences, Center for Health Sciences Research, Universidad Adventista del Plata, Libertador San Martín, Entre Ríos, Argentina; ^2^Institute for Food Science and Nutrition, Universidad Adventista del Plata, Libertador San Martín, Entre Ríos, Argentina; ^3^Center for Child Nutrition Studies Dr. Alejandro O'Donnell (CESNI), Ciudad Autónoma de Buenos Aires, Argentina; ^4^School of Public Health, Center for Nutrition, Healthy Lifestyle and Disease Prevention, Loma Linda University, Loma Linda, CA, United States

**Keywords:** 24-h dietary recall, nutritional assessment, open-access tool, AMPM, food chemical composition, Argentina, Spanish

## Abstract

**Background:** Latin American countries show a fast-growing rate of non-communicable diseases (NCDs) and diet is a critical risk factor that must be properly assessed. Automated dietary assessment tools to collect 24-h dietary recalls (24HR) are lacking in Argentina.

**Objective:** This study aimed to develop an open-access automated tool (MAR24) for collecting 24HR using a multiple pass method and a database containing foods and recipes commonly consumed in Argentina.

**Methods:** MAR24 was developed based on data from 1,285 24HR provided by male and female participants aged 18 to 68 years from the six Argentinian geographical regions. The main structure and interface of the tool were designed using Visual Basic for Applications programming language in Excel Microsoft Office 365, integrating the five steps of the United States Department of Agriculture (USDA) Automated Multiple-Pass Method (AMPM) for the application of 24HR in Spanish. The tool underwent alpha testing and expert assessment to address structural and usability issues. Critical feedback and face validation from researchers and experienced dietitians, and repeated testing to collect 24HR were used to adjust and improve the tool.

**Results:** A total of 968 food items and 100 standard Argentinian recipes were added to its database. MAR24 allows the estimation of the nutrient profile of dietary intake. The analytic food composition includes energy and 50 nutrients including water, macronutrients, total dietary fiber, total sugar, 10 minerals, 19 vitamins, eight fatty acids, cholesterol, ethyl alcohol, caffeine, and theobromine. MAR24 includes a user manual and technical manual to guide users to apply changes (e.g., add foods or recipes, or change food designation according to local terms) to fit different research and clinical applications.

**Conclusions:** MAR24 is the first tool that uses the AMPM methodology for 24HR applications in Argentina. The tool may be used in clinical practice and clinical trials for monitoring purposes, and in validation of food frequency questionnaires (FFQ) for nutritional epidemiology studies addressing dietary-associated risk factors for NCDs.

## Introduction

Diet is a variable of interest when investigating chronic disease etiology. Foremost among the dietary assessment methodologies utilized in population studies are the 24-hr dietary recall, food diary or record, and food frequency questionnaire. While each of these methods has its strengths and limitations, the 24-h dietary recall (24HR) is often the method of choice when respondent burden is a concern, such as in populations with limited literacy or patience and motivation to comply with the rigors of food recording (1, 2). In conducting 24HR, detailed information on food, drinks, and supplements consumed during the past 24 h (3, 4) is collected via interviews or through self-report. A single 24HR may be used in clinical practice and research to measure a day's food, nutrient or energy intake but multiple 24 HRs would be needed to estimate usual or habitual intake (5, 6), the exposure that is often associated with health outcomes (7, 8). Since 24HR allows a high level of specificity due to open-ended food lists and detailed food preparation and description, and uses short-term, episodic memory that is less subject to recall bias than the memory used for food frequency questionnaires (FFQ), multiple or repeated 24 HRs are particularly useful as a reference standard for validating FFQ, the less-costly dietary assessment method used in large epidemiological studies to estimate habitual diet (7, 8).

Assessing dietary habits is important since these habits impact non-communicable disease (NCD) development and mortality (5, 9–11). Studies carried out in the Argentine population by our group and others have found that the majority of this population does not meet the nutritional recommendations of national health agencies, particularly the intake of fruits, vegetables, whole grains, legumes, nuts, and seeds (12–15). NCDs are known to be the leading causes of death in the world, disproportionately affecting medium- and low-income countries where half of the premature deaths from NCDs occur (9, 10). According to the World Health Organization (WHO), nearly 80% of the total deaths in Argentina are associated with NCDs (10) while unhealthy eating habits rank second among the ten risk factors associated with disabilities and deaths (11). The other main risk factors for NCDs, such as elevated fasting blood glucose, high body mass index, high blood pressure, and dyslipidemia are also diet-related (5, 9, 10). These data indicate the need for accurate assessment of dietary exposure in observational and intervention studies that investigate the role of diet in NCDs. However, there is still a lack of tools to facilitate the collection of reliable information on food consumption in Argentina and other Latin America countries.

The need for effective ways to collect nutritional data and estimate food consumption at the population level paved the way for the development of technology-based dietary assessment tools for various population groups (e.g., age, country/language) (16–18). Most 24HR applications, in particular, are based on the automated multiple-pass method (AMPM) approach, a reliable 24HR collection method designed by the United States Department of Agriculture (USDA) to reduce misreporting or underreporting bias (19, 20). In general, the applications are either self-administered, where a participant completes the recall in the absence of a researcher (21–31), or interviewer-administered, where the interviewer uses the application to collect/analyze dietary recall data in the presence of a participant (32–38).

Two interviewer-administered tools have been developed and used among Spanish-speaking adults: the bilingual interactive multimedia diet recall tool for low-literacy Hispanic population in the US (39), and the GloboDiet for the Mexican population (40). Argentina, however, has yet to develop a culturally-specific dietary assessment tool using locally-named foods, and that integrates all the steps of a 24HR using the AMPM and systematizes the assessment of a dietary intake. Therefore, we developed an automated interviewer-administered tool for collecting Multiple-Pass 24HR containing foods and recipes commonly consumed in Argentina (MAR24). The tool was devised to be available as an open-access source and with the possibility of being expanded with the addition of foods and recipes for fitting different research and clinical applications.

## Materials and Methods

MAR24 is an open-access tool whose main structures and interfaces were designed using Visual Basic for Applications programming language in Excel Microsoft Office 365. MAR24 was developed in the Spanish language and was designed to be operated by trained nutrition professionals for an interviewer-administered application of the multiple-pass 24HR. The tool integrates the five steps of the AMPM (19, 20), along which respondents receive cues to assist them to remember and describe the foods consumed the previous day (the preceding 24 h). The steps include: (1) “*quick food list,”* which involves listing foods and beverages consumed during the previous day; (2) “*forgotten foods,”* inquiry is made for foods possibly forgotten during the quick list; (3) “*time and occasion*,” adding the time and eating occasion for each food: (4) “*detailed cycle*,” a detailed description, amount, and additions for each food is done, then the 24-h day is reviewed; and, (5) “*final probe*,” a final inquiry for anything else consumed is done. The tool was developed over approximately 30 months. A user's instruction manual and a technical manual, as well as a how-to-use tutorial video in Spanish, were created for MAR24. The technical manual provides instructions for: adding, modifying, or deleting foods; creating recipes; and, modifying food names according to language variations. A visual aid for the portion size references goes with the tool and can be provided to the respondent/interviewee. The tool and its manuals can be downloaded from the website http://investigacion.uap.edu.ar/MAR24-Argentina. Users may register to receive notifications and updates about the tool.

### Creation of the MAR24 Database

MAR24 was initially constructed using 120 audio-recorded 24HR collected via telephone interviews. Improvements and adjustments to the tool resulted in the expansion of its database. A total of 1,165 24HR served as the basis for identifying the foods and recipes to be included in the MAR24 database. Male and female participants aged 18–68 years from the six Argentinian regions—Central (*n* = 237), Northwest (*n* = 140), Northeast (*n* = 191), Buenos Aires (*n* = 267), South (*n* = 170), and Cuyo (*n* = 160)—provided these 24HR through recorded telephone interviews.

The Food Analysis and Registration System from the Ministry of Health of Argentina, “*Sistema de Análisis y Registro de Alimentos*” (SARA) Version 1.2.29 (41) was utilized to build the database of food items and standard recipes for MAR24. The database includes simple foods consisting of a single ingredient (e.g., vegetables, fruits, nuts, milk) and complex foods containing a few ingredients (e.g., cakes, biscuits, bread). These simple and complex foods were defined as *single food* items. SARA allows the use of two types of recipes: *standard* and *compound*. *Standard* recipes are traditional preparations of Argentine cuisine commonly mentioned in the 24HR and extracted from the SARA. Although the standard recipes have predefined ingredients, there is an option to include or exclude ingredients. *Compound* recipes describe meals reported during the interview, but which do not currently exist in the database. Their content is added using details provided by the interviewee. For example, report of a vegetarian dough recipe would not facilitate use of the existing recipe (which includes animal fat). Amendments to this compound recipe can be made using the tool to select the appropriate plant-based fat. Also, if the interviewee mentions allergy to a specific type of food and reports the consumption of a different version of a standard recipe, this may be created as a compound recipe. Both *simple* and *complex* foods can be linked to recipes. This feature allows interviewers to specifically input information that reflects diversity of eating preferences and needs. Modifications, additions, and deletions of foods and recipes are included in the user manual. All modifications remain in the database of the user once the tool is downloaded on a device.

### Amount and Portion Sizes

**Figure 1:**
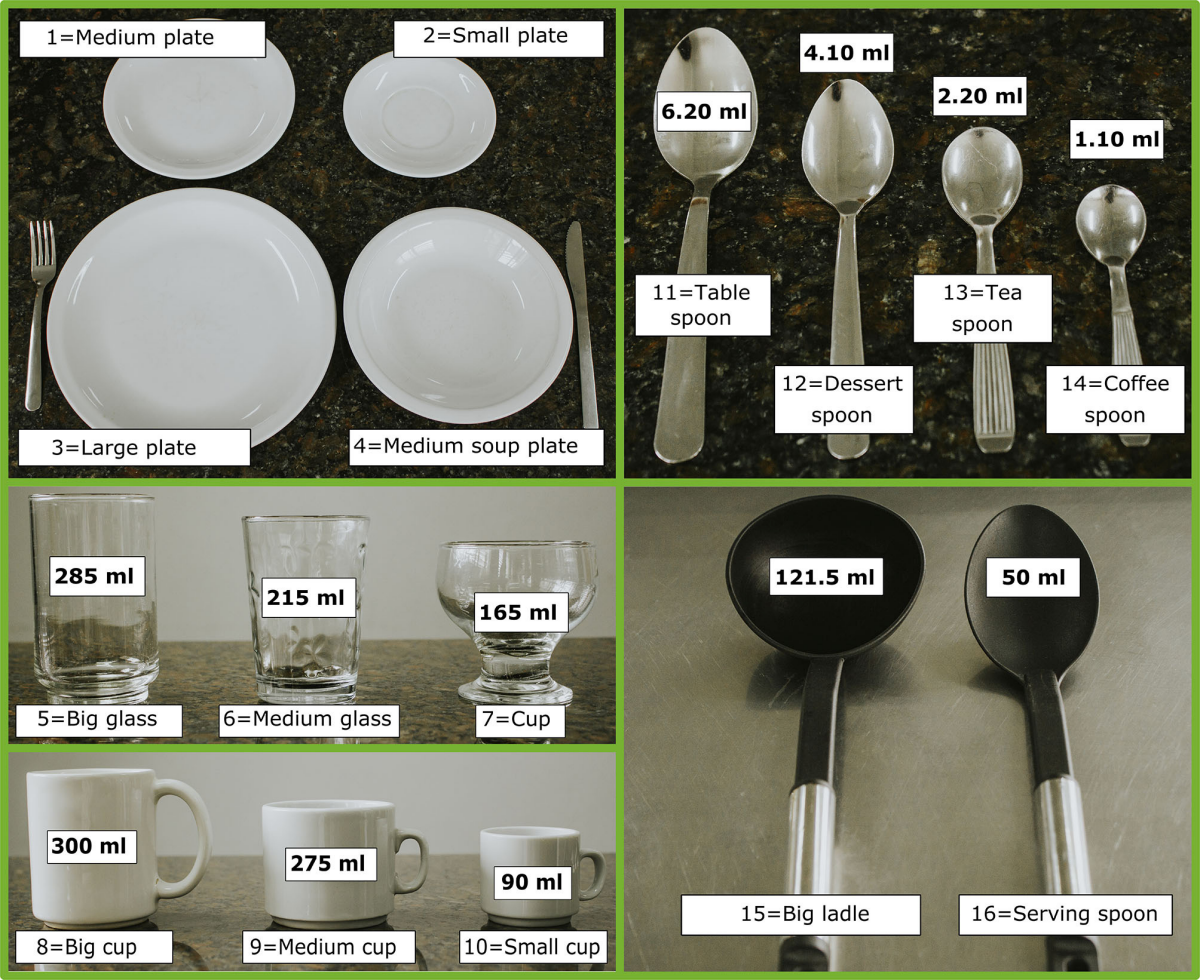
Household measures references of the MAR24 photo album.

The most common utensils and household measures used in Argentina were identified based on the information of the initial 120 24 HRs collected through telephone interviews with no visual aid from 6 Argentinian regions (center, northwest, northeast, Buenos Aires, south, and Cuyo). From this information, a visual aid containing photographs of 16 common household measures was constructed (Figure 1) to serve as portion size reference during the application of 24HR.

A database of weight equivalents of household measures, portions and units was built and correspond to the “equivalences sheet” of the tool. For most items (*n* = 781), such weight equivalents were extracted from literature sources (41–43). The remaining measurements were either weighted or estimated by the following procedure: (1) foods with similar density and/or characteristics were grouped, for example, corn flour, wheat flour, rice flour, etc. were put under the “flour” group, while olive oil, corn oil, sunflower oil, etc. were grouped under “oils”; (2) weights of 2–6 foods from each group were measured in the 16 utensils; and (3) corroborated with the measures and weights of foods reported in Argentina (41, 42). A total of 100 food items, in 16 different utensils (1,600 weights), were measured in the Laboratory of Nutrition and Dietetics by two researchers using Aspen model precision balances for small and large volumes (MH-500, range 0.1 to 500 g and EK3052, range 1 g to 2 kg). The results served as a basis to establish the proportionality relations of weight between the different household measures for each food group. The weight equivalents of 87 food items were estimated according to the measurements of the representative items from each food group. A bivariate correlation test was performed between the weights of the foods in the same group resulting in correlation (*r*) values ≥0.99 (*p* < 0.0001). The complete list of foods and their weights in the different household measures is found in the technical manual of MAR24.

Standardized portion weights for preparations such as a piece of cake or a slice of pizza were obtained from the SARA program. Some fruits and vegetables have weights per unit for different sizes (small, medium, and large), when appropriate.

### Nutrient Profile of Foods

The nutrient profile of all food items was taken from the USDA Food Composition Databases (43), except for 5 particular foods (*Amargo serrano, Amargo serrano diet, Bizcochos de grasa, Chipá* and *pan criollo*) for which the composition was obtained from the SARA program (41). Each food item selected from the USDA database has its nutrient composition (water, energy, fiber, macro- and micronutrients) and ingredient(s) (when applicable) compared and checked for similarity with data from the following available Argentinian databases: SARA (41), ArgenFood Food Composition Table (44), Nutrinfo database (45), or from the Central American Food Composition Table of the Institute of Nutrition of Central America and Panama (INCAP) (46). For cooked foods, all cooking methods were searched for each food and those used in Argentina were selected. As there is no information on cooked foods from local databases for comparison, cooked versions of the raw foods from the same USDA data source (Legacy, Survey, or Foundation) were selected (43).

### Quality Control of the Tool

The initial version of MAR24—developed from audio-recorded 120 dietary recalls performed by five trained interviewers using the AMPM methodology—underwent alpha testing to address structural and usability issues. During the entry of the 24 HRs onto the MAR24, interviewers provided weekly reports of their experiences and discussed their difficulties and suggestions for adjustments to the tool with the researchers. In addition, two researchers independently compared the data that was entered onto the MAR24 system by the five interviewers against the audio records of the recalls to check for data entry accuracy.

After completing the entry of 1,165 additional 24 HRs sometime in 2020, seven dietitians/experts from different states of Argentina with experience in the field of nutritional assessment and research were invited to assess the MAR24. A questionnaire with nine Likert-type scale and three open-ended questions was used for the evaluation. The dietitians/experts were asked whether the tool was intuitive, comprehensive, user-friendly, suitable, and if it was effective to optimize the dietary data collection for 24HR applications in the context of research and/or professional practice. The open-ended questions pertained to observations and suggestions for improvement of the sections on interviewer and respondent information, available foods and recipes, portion sizes, utensils and household measures, nutrient composition database, layout, and design.

### Ethical Aspects

The MAR24 tool was developed in the context of an ongoing research study that had been evaluated and approved by the Research Ethics Committee (resolution N° 1.7-8/2016), affiliated to the National Registry of Health Research (N° 237), Ministry of Health, Argentina. Participants were included in the study by invitation and acceptance of the terms of the Informed Consent.

## Results

The current MAR24 version contains a total of 968 food items including simple and complex food and 100 standard recipes commonly consumed in Argentina and 50 nutrients and other food components in its database. The tool is open access whose main structures and interfaces are in Excel Microsoft Office 365.

### Background Capture Information

**Figure 2:**
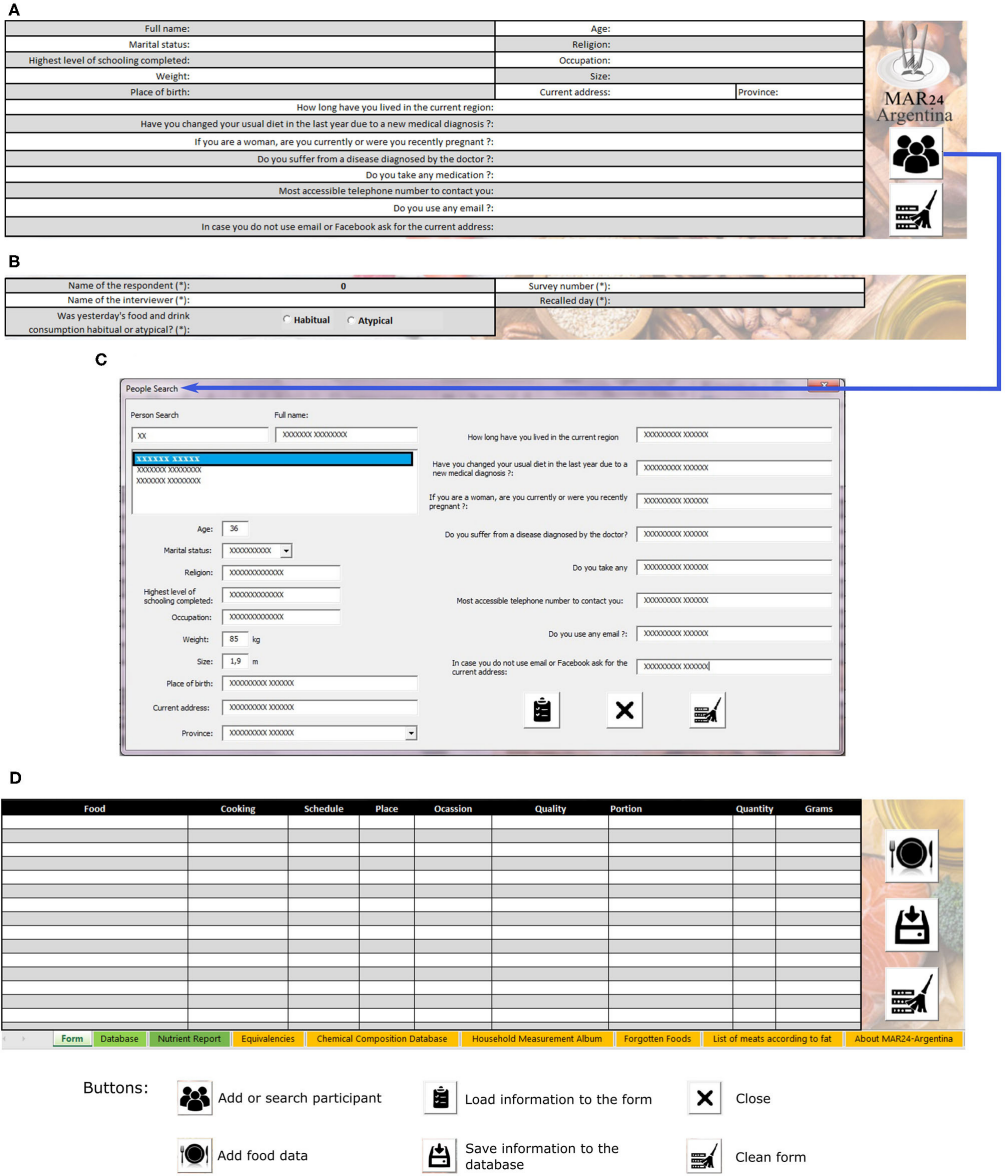
Sections of the main form of the MAR24. **(A)** Interviewee data module with sociodemographic, health, and contact information; **(B)** Identification section of the 24-h dietary recall and interviewer; **(C)** Module for adding or searching participants; **(D)** Food data module resulting from the 24HR.

During the first interview step, information on the interviewee, the interviewer, and the recall day (date and whether intake is considered habitual or atypical by the respondent) is collected (Figures 2A,B). The button to add or search participant opens the screen shown in Figure 2C, which has a search engine for information stored in the database along with three options of controls to transfer information to the form, to close, or to delete the data.

### Dietary Intake Capture Information

Figure 2D shows the section intended for managing food data from the 24HR and lists the foods consumed according to the loading order. This section has three buttons to either open the module to add food data, transfer the information to the database, or delete the data.

**Figure 3:**
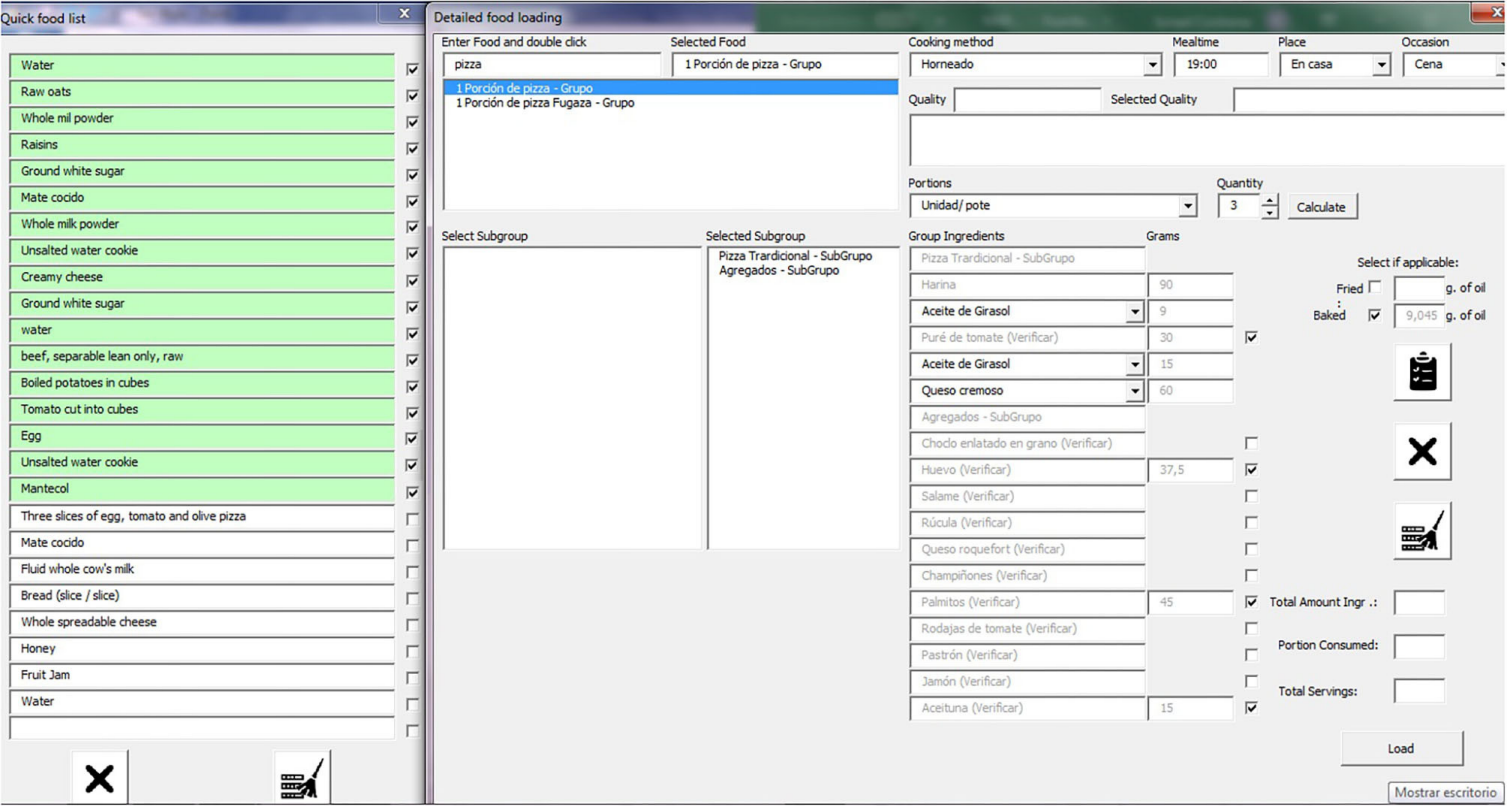
Dietary intake module of the MAR24.

Figure 3 shows the entry fields window to input foods reported to have been eaten over the past 24-h period through the steps of the AMPM. During the “quick food list” step, the interviewer enters all foods, recipes and beverages from the “quick list items,” with no additional details or quantification. The interviewer checks the completeness of the “quick food list” focusing the respondent's attention on some food groups that are often forgotten, such as sweets, sodas, snacks, dressings, broths, condiments, and others. The “time, place and occasion” step is used to indicate the time, the place where the meal was eaten (e.g., home, restaurant, family home), and the eating occasion (e.g., breakfast, lunch, snack, dinner).

The “detailed food loading” cycle is used to load a complete description of each food or recipe from the quick list into the tool using an intelligent search in the database. During this step, ingredients may be excluded from standard recipes, if needed. Furthermore, some foods with different options have a drop-down list to choose the appropriate option (e.g., options for milk are milk powder, whole milk, soymilk). Foods and recipes have local names (Argentine), including vegetarian and vegan dishes.

Determination of the appropriate foods is optimized by having a selection of several forms of a specific food. For example, carrots can be “cubed carrot, sautéed with oil and salt” or “raw grated carrot,” etc. Alternatively, each food may be loaded separately, and the cooking method selected accordingly (e.g., fried, baked, cooked or boiled, steamed or sautéed). When selecting the fried or baked option, an amount of oil corresponding to 10 and 3%, respectively, of the total weight of the food will be added automatically to the list, indicating the absorption of oil according to the cooking method (41). In the field dedicated to food quality, the interviewer can type-in additional details about the food that are not reflected in the automated list (e.g., lactose-free).

The foods that include certain cooking methods in their description are defined based on the information provided by the USDA. Cooked and raw items are selected from the same database (i.e., Survey, SR Legacy). Although we sought to introduce in the tool as many cooking options as possible for each food, the USDA database has only one cooking method for some foods.

The quantity of foods and beverages consumed is chosen with the aid of a photographic album of the 16 household measures and utensils (Figure 1). A fraction of the utensil may be used to quantify the amount of food consumed or the food unit. A household measure will appear in the list if the corresponding weights are available in the MAR24 “*Equivalencies*” sheet. The quantity is chosen according to the selected utensil or unit. By pressing the “*calculate*” button, the corresponding weight in grams will show up. Food quantity loading is in grams and net weight. Liquid quantities and measures are indicated in milliliters (mL), and supplements in milligrams (mg), micrograms (μg), or International Units (IU).

There is a section to calculate the amount of food consumed from a non-standard compound recipe. For example, “The participant prepared a salad for five people using four tomatoes. He reported having consumed two servings of the salad. How many tomatoes did the participant eat?” The following equation is used to determine the amount of food consumed:

$$Quantity=\;\frac{Portion\;consumed}{Total\;portions}*Total\;amount\;of\;ingredient$$

Where:

*Quantity* = *Amount of food consumed*

*Portion consumed* = *Number of portions consumed by the participant*

*Total portions* = *Number of portions prepared*

*Total amount of ingredient* = *Total amount of added ingredient(s)*.

For each entered food, the button to send information to the form is pressed and the corresponding box to that food in the quick list is checked. If the respondent does not provide enough information about a meal or preparation, the interviewer should delve further and ask additional questions until the required level of description is obtained.

To end the survey, the “final probe” step is carried out, verifying that all the foods mentioned in the quick list are included, and encouraging the respondent to remember any missing item even if consumed in small quantities. The forgotten food is then added to the quick list and detailed loading is carried out.

### Processing and Analysis of Nutritional Information

At the completion of food intake loading, all data obtained in the 24HR will be shown in Figure 2D. Once the information is in the database, modifying a specific food will entail deleting the record (row) from the database and reloading the food, then sending it back to the database. The tool has a button to assess the nutrient profile of the food intake inside the “database” section. The energy and nutrient report includes 50 nutrients and other food components: water, macronutrients (carbohydrates, proteins including from vegetable and animal sources, lipids), ash, total dietary fiber, total sugar, 10 minerals (calcium, iron, magnesium, phosphorous, potassium, sodium, zinc, copper, manganese, selenium), 13 vitamins (ascorbic acid, thiamin, riboflavin, niacin, pantothenic acid, pyridoxine, folate, choline, vitamin B12, vitamin A, retinol, beta-carotene, alpha-carotene, alpha-tocopherol, vitamin D, Vitamin K), 8 fatty acids (saturated, monounsaturated, polyunsaturated, n-3 18:3, n-3 20:5, n-3 22:5 n-3 22:6, n-6 18:2), beta-cryptoxanthin, lycopene, lutein, zeaxanthin, cholesterol, ethylic alcohol, caffeine, and theobromine. Table 1 presents an example of the output, showing the nutrient report totals of a 24HR loaded into MAR24 that reflects Table 2.

**Table 1:** Example of nutrient totals report of a 24HR loaded into MAR24.

**Name**	**Amount**	**Unit**
Water	1903.100	mL
Energy	2131.090	kcal
Protein	89.604	g
Total lipid (fat)	90.997	g
Ash	8.084	g
Carbohydrate, by difference	238.448	g
Fiber, total dietary	11.755	g
Sugars, total including NLEA	79.564	g
Calcium, Ca	1331.836	mg
Iron, Fe	15.306	mg
Magnesium, Mg	475.152	mg
Phosphorus, P	1421.732	mg
Potassium, K	2651.657	mg
Sodium, Na	1613.296	mg
Zinc, Zn	13.944	mg
Copper, Cu	1.128	mg
Manganese, Mn	1.208	mg
Selenium, Se	133.513	μg
Vitamin C, total ascorbic acid	32.895	mg
Thiamin	1.810	mg
Riboflavin	2.841	mg
Niacin	34.814	mg
Pantothenic acid	3.535	mg
Vitamin B-6	1.613	mg
Folate, total	376.745	μg
Choline, total	457.463	mg
Vitamin B-12	6.730	μg
Vitamin A, RAE	618.622	μg – RAE
Retinol	543.216	μg
Carotene, beta	778.990	μg
Carotene, alpha	143.701	μg
Cryptoxanthin, beta	8.139	μg
Lycopene	4414.760	μg
Lutein + zeaxanthin	671.352	μg
Vitamin E (alpha-tocopherol)	9.208	mg
Vitamin D (D2 + D3), International Units	250.703	μg
Vitamin K (phylloquinone)	30.257	μg
Fatty acids, total saturated	33.616	g
Fatty acids, total monounsaturated	36.752	g
Fatty acids, total polyunsaturated	13.063	g
Omega 3 (18:3)	0.554	g
20:5 n-3 (EPA)	0.007	g
22:5 n-3 (DPA)	0.018	g
22:6 n-3 (DHA)	0.038	g
Omega 6 (18:2)	11.892	g
Cholesterol	482.997	mg
Alcohol, ethyl	0.000	g
Caffeine	624.000	mg
Theobromine	0.000	mg

**Table 2:** Sample of food list from a 24HR loaded into MAR24.

**Food**	**Schedule**	**Place**	**Occasion**	**Portion**	**Quantity**	**Grams**
Water (mL)	7:30:00	At home		1 Big cup (8)	1	300.0
Raw oats	8:00:00	At home	Breakfast	1 Tablespoon (11)	3	18.0
Whole milk powder	8:00:00	At home	Breakfast	1 Teaspoon (13)	2	4.0
Raisins	8:00:00	At home	Breakfast	Medium unit	10	4.0
Ground white sugar	8:00:00	At home	Breakfast	1 Coffee spoon (14)	1	2.0
Brown coffee (mL)	8:00:00	At home	Breakfast	1 Big cup (8)	1	300.0
Unsalted water cookie	8:00:00	At home	Breakfast	Unit / pot	5	30.0
Creamy cheese	8:00:00	At home	Breakfast	1 Teaspoon (13)	5	41.1
Ground white sugar	8:00:00	At home	Breakfast	1 Coffee spoon (14)	2.5	5.0
Water (mL)	9:30:00	At home		1 Big glass (5)	1	285.0
Beef, separable lean only, raw	13:00:00	At home	Lunch	Medium unit	1	110.0
Boiled potatoes in cubes	13:00:00	At home	Lunch	1 Big ladle (15)	1	103.0
Tomato cut into cubes	13:00:00	At home	Lunch	1 Big ladle (15)	1	82.0
Egg	13:00:00	At home	Lunch	Unit / pot	1	42.0
Unsalted water cookie	13:00:00	At home	Lunch	Unit / pot	2	12.0
Mantecol (portion)	13:00:00	At home	Dessert	Unit / pot	1	20.0
Three slices of pizza (egg, tomato and olives)	19:00:00	Out of home	Dinner	Unit / pot	3	301.5
Water (mL)	19:00:00	Out of home	Dinner	1 Big glass (5)	1	285.0
Brown coffee (mL)	19:00:00	Out of home	Dinner	1 Big cup (8)	1	300.0
Fluid whole cow's milk (mL)	19:00:00	Out of home	Dinner	1 Tablespoon (11)	2	12.4
Bread (slice / slice)	19:00:00	Out of home	Dinner	Unit / pot	4	48.0
Whole spreadable cheese	19:00:00	Out of home	Dinner	1 Coffee spoon (14)	4	7.9
Honey	19:00:00	Out of home	Dinner	1 Teaspoon (13)	2	12.6
Fruit jam	19:00:00	Out of home	Dinner	1 Teaspoon (13)	2	10.0

**Figure 4:**
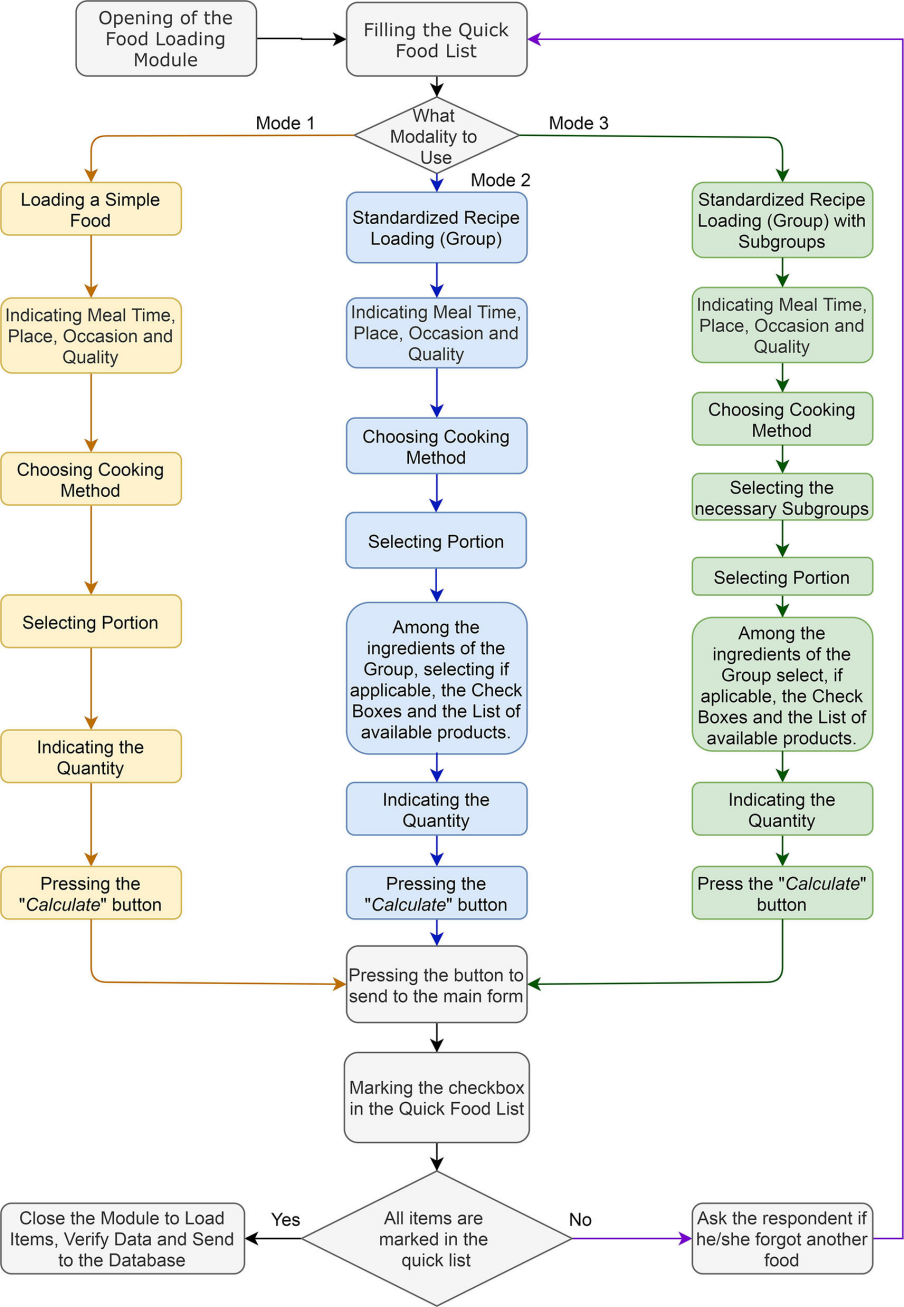
Loading food flowchart of the MAR24.

A food loading flow diagram is summarized in Figure 4. Due to the need to apply multiple 24HR to the same person, the names of the participants are recorded in a database. To maintain confidentiality when analyzing the data in the nutrient report section, the MAR24 can replace the participant's name with a code based on the record of each 24 h and the questionnaire number.

### Assessment of the Tool

The initial version of the tool was constructed based on the alpha test of 120 24HR. The subsequent application of 1,165 24HR allowed a dynamic improvement of the tool as observations and suggestions were pointed out by researchers while conducting the dietary recalls. The tool underwent adjustments to facilitate the implementation of the five steps of the AMPM. The database of foods and standard recipes expanded due to food items reported in the 24HR. The tool was checked for accuracy in processing data. Likewise, the algorithms were improved optimizing the response time of the program in estimating energy intake and the nutrient composition of foods. Additionally, it was confirmed that the smart search engine obtained the most updated user information, and that no food was left in the database outside of the search. Improvements were also made in the interface to make the tool user-friendly.

The seven external dietitians “agree” or “totally agree” with the easy handling, usefulness, and comprehensiveness of MAR24 as an application for dietary recalls and its efficiency in processing 24HR data. However, they “neither agree nor disagree” on the “intuitive aspect of the tool” (Supplementary Figure 1). Feedback for improvement of MAR24 from the same expert group resulted in expanding the nutrient composition database by adding more nutrients, for example, n-3 fatty acids, and providing the option to add the exact measure of food in grams or milliliters aside from the household measures (Supplementary Figure 2). The instructional information found in the technical and user manuals were correspondingly completed.

## Discussion

MAR24 is the first tool that uses the AMPM methodology for an automated application of 24HR in Argentina. The tool was developed to be culture-specific by including commonly consumed foods in their local names from the six regions of Argentina and to optimize the dietary assessment process by improving the accuracy of collecting dietary data and reducing recall bias. The tool was designed to be flexible so its food and nutrient database can potentially expand, and open access so it would be useful in different research and clinical settings. The food and nutrient composition database of MAR24 includes SARA, the USDA Food Composition Database, and other databases.

The collection and processing of human dietary intake are complex tasks (47). Because of this, modern technology had been utilized to develop more efficient dietary assessment approaches to assess the dietary intake of individuals and groups and provide a comprehensive system of data collection, coding, and intake calculation (5). Such methods are needed for clinical and epidemiological investigations, since they improve the quality and reliability of the information obtained, reducing possible errors associated with manual coding, as well as optimizing resources for information processing (6).

Not many interviewer-administered automated tools have been described in the literature for administering 24HR to adults from different parts of the world. Two were designed for Korea (33, 36), one for India (specifically New Delhi, Mumbai, and Trivandrum) (37), one for Brazil (34), one for Serbia and Balkan region (35), one for the US low-literate Spanish- and English-speaking citizens (39), and one for the European Union (32). The latter, originally called EPIC-Soft (32) has been renamed to GloboDiet and adapted to the Brazilian and Mexican populations (40). There is no existing automated tool to collect 24HR in Argentina. The development of the MAR24 tool is an option to meet this current need. While other tools could potentially be adapted for use in Argentina, the lack of a national food composition database (48) is a present limitation. The only food composition table generated on a national scale in Argentina was developed in the mid-1900. Later, some compilations were produced by the University of Cordoba and the National University of La Plata using the very first food composition table, individual publications of laboratories, research groups, and foreign nutritional databases. More recently, an *ad hoc* food composition table was prepared for the analysis of nutrient intake for the first National Nutrition and Health Survey in 2004/2005, and its data was incorporated into the SARA software. In 2015 a systematic review searched for newer information to expand the current food composition table of Argentina and collected individual food items and multi-ingredient foods (49).

In our study, the substantial number of 24HR collected from all six regions of Argentina allowed us to identify and include into the MAR24 database a greater number of foods, preparations, and recipes than those reported by the SARA (41), a free access system containing the chemical composition of 379 foods commonly consumed in Argentina. However, it should be noted that food monotony is a reported challenge associated with the usual dietary intake in Argentina (14). For this reason, the MAR24 food database is equipped with fewer food items (*n* = 968) than other tools, which usually have more than 1,000 items (18).

The use of the USDA food composition tables for the construction of MAR24 is justified because of the lack of a national database (48). In addition, this offers the possibility of expanding nutrient reports. The use of the USDA database in MAR24 allowed the generation of more detailed nutritional chemical composition information than would be possible using other databases. As a result, the MAR24 has 50 elements available in the chemical composition table in contrast with around 25 elements currently found in the database SARA (41).

To minimize biases in the quantification of bioactive and other food compounds in the MAR24, the composition of each selected food from the USDA was compared with the tables of food composition from SARA, ArgenFoods, Nutrinfo, and the Central American Food Composition Table of the Institute of Nutrition of Central America and Panama (INCAP). For each food, a non-fortified or a fortified version was sought seeking to match the consumption in Argentina. After obtaining one or more options for each food, its composition was compared with local data. The food items that were most similar in description and nutrients were selected. This procedure was applied for the selection of both raw and industrialized foods. For foods prepared by simple food processing (e.g., boiled, sauté, baked, etc.), for which there was no local composition information for comparison, we selected the processed version of a corresponding raw food within the same USDA database source (Legacy, Survey or Foundation) (43). All possible cooking methods were searched for each food and those used in Argentina were selected.

The USDA Food Survey Research Group has developed automated methods to assess dietary intake using standardized procedures and streamlining the availability of survey results (47). The USDA dietary intake instrument incorporates the AMPM which is one of the most widely used and comprehensive methods used for collecting dietary recall data nowadays (50). The MAR24 standardizes 24HR collection using the AMPM steps (20), thus increasing the quality of data collection. This procedure would not be possible with the use of SARA (41), since it does not provide the systematic structure to administer 24HR.

Trained interviewers are required for the proper conduct of 24HR (4, 6, 51, 52). They can be applied remotely through phone calls or through in-person interviews (53). MAR24 assists the interviewer to guide the participant in recalling and providing specific and detailed description of the foods and beverages consumed during the previous day, including their quality (types of food, cooking method, recipe ingredients, trade names, and others). If used to assess or estimate habitual intake, multiple 24 HRs need to be collected; if unannounced, respondents will be prevented from modifying their habitual consumption which can then reduce bias (5).

Reliable estimates of the amount of food consumed in a 24HR must be supported by visual resources, such as photographic atlases, images of household measures, or food models (3, 4, 6). Weights and portion sizes of food amounts in the MAR24 are determined based on the portion size visual aid (Figure 1). During the administration of MAR24 to collect 24HR, it is recommended that both the interviewer and the respondent have the portion size visual aid (54).

Automated collection of dietary intake data maintains consistency in all the phases of the interviews and a systematic data entry (50) which improves the accuracy of food and nutrient intake estimates (55). For 24HR data collection, automated methods also avoid biases that may occur during the manual loading of information, whether it is the omission or inappropriate addition of data, imprecise estimation of portions and weights, coding failures, and gross weight and net weight problems, among others (3, 55). These new technology-based tools are considered appropriate instruments for nutrition surveillance, as they are efficient in terms of both data collection (front end) and analysis (back end) for the study staff. As an automated method, the MAR24 may be used to collect dietary data in epidemiological studies combined with other instruments such as FFQ (22, 56). The instrument may also be useful in clinical practice by health professionals, mainly dietitians, to control the consumption of food and beverages of patients or to perform food recall.

Latin America has one of the fastest-growing rates of NCDs (57), despite that, a systematic scoping review showed that only about 0.5% of all health intervention studies targeting multiple risk factors for chronic diseases were done in South America (58). There is a critical need for more studies to address NCDs in Latin America. The use of automated tools to assess dietary intakes such as the MAR24 and other dietary assessment approaches may be helpful for the advancement of nutritional research and to implement tailored health interventions. Therefore, the tool may be used to assess food intervention studies addressing risk factors for NCDs (10).

## Limitations

The usefulness and comprehensiveness of this tool have been tested but its performance in analyzing dietary intake is yet to be compared with other similar tools that employ the AMPM method and the USDA nutrient database (39). The use of the MAR24 tool requires a license of Excel Microsoft Office, which is widely used but may not be available on some systems. Despite its intended use in Argentina, one of MAR24's limitations is the heavy reliance on the USDA food composition database. This is because the current food chemical composition tables of Argentina are still limited in scope and undergoing slow development (48). Food composition may vary depending on the characteristics of the soil, cultivars, season, fortification uses, and many other aspects, thus the chemical composition of foods grown in Argentina may not be accurately reflected by the chemical composition estimates for the same foods on the USDA database. However, developers of the Argentinian software SARA also acknowledged in its program the use of information from the USDA food database and other foreign databases. Therefore, the MAR24 may contribute significantly to the field of nutrition, although with its limitations, considering the demands for studies addressing dietary risk factors for NCDs in Argentina.

## Conclusions

This study presents the MAR24 as the first tool that uses the AMPM methodology for the automated collection of 24HR in Argentina. This dietary data collection tool includes 968 foods, including 100 recipes that represent foods and recipes from the six regions of the country and 50 nutrients and other food components. The food database uses local names for food and recipes, a visual aid for portion size estimation, and is freely accessible to researchers and health professionals. The MAR24 may be used to optimize dietary data collection and nutrient consumption analyses in clinical practice and clinical-trials for monitoring purposes and the validation processes of food frequency questionnaires (FFQ) for nutritional epidemiology studies addressing dietary-associated risk factors for NCDs.

## Data Availability Statement

The raw data supporting the conclusions of this article will be made available by the authors, without undue reservation.

## Ethics Statement

The studies involving human participants were reviewed and approved by Research Ethics Committee, affiliated to the National Registry of Health Research (N° 237), Ministry of Health, Argentina. The patients/participants provided their written informed consent to participate in this study.

## Author Contributions

SP, FP, and IC-G participated in the design of the study. IC-G and DX participated in the programming and development of the tool. SL, RG, and BC participated in data collection. MM, MZ, GS-S, FP, IC-G, DX, SL, RG, BC, and SP participated in data analyses and evaluation of the tool. IC-G, SP, FP, MZ, GS-S, MM, JS, SL, RG, and BC provided critical comments and worked in manuscript preparation. All authors reviewed the content of the manuscript and approved the final version.

## Conflict of Interest

The authors declare that the research was conducted in the absence of any commercial or financial relationships that could be construed as a potential conflict of interest.

## Publisher's Note

All claims expressed in this article are solely those of the authors and do not necessarily represent those of their affiliated organizations, or those of the publisher, the editors and the reviewers. Any product that may be evaluated in this article, or claim that may be made by its manufacturer, is not guaranteed or endorsed by the publisher.

## Supplementary Material

Supplementary Figure 1 Quantitative experts' assessment on the MAR24 tool.

Supplementary Figure 2 Qualitative experts' assessment on the MAR24 tool.

## Abbreviations

AMPM: automated multiple-pass method
MAR24: *Método Automatizado de Recordatorio de 24 horas* (Automated 24-h recall method)
NCDs: non-communicable diseases
24HR: 24-h dietary recall
SARA: *Sistema de Análisis y Registro de Alimentos*
USDA: United State Department of Agriculture
WHO: World Health Organization.
