# Supplementary figures and images for: Development and Usability Study of an Open-Access Interviewer-Administered Automated 24-h Dietary Recall Tool in Argentina: MAR24
Source: Front Nutr. 2021 Aug 5;8:642387. doi: 10.3389/fnut.2021.642387 (PMC8374600; doi:10.3389/fnut.2021.642387)

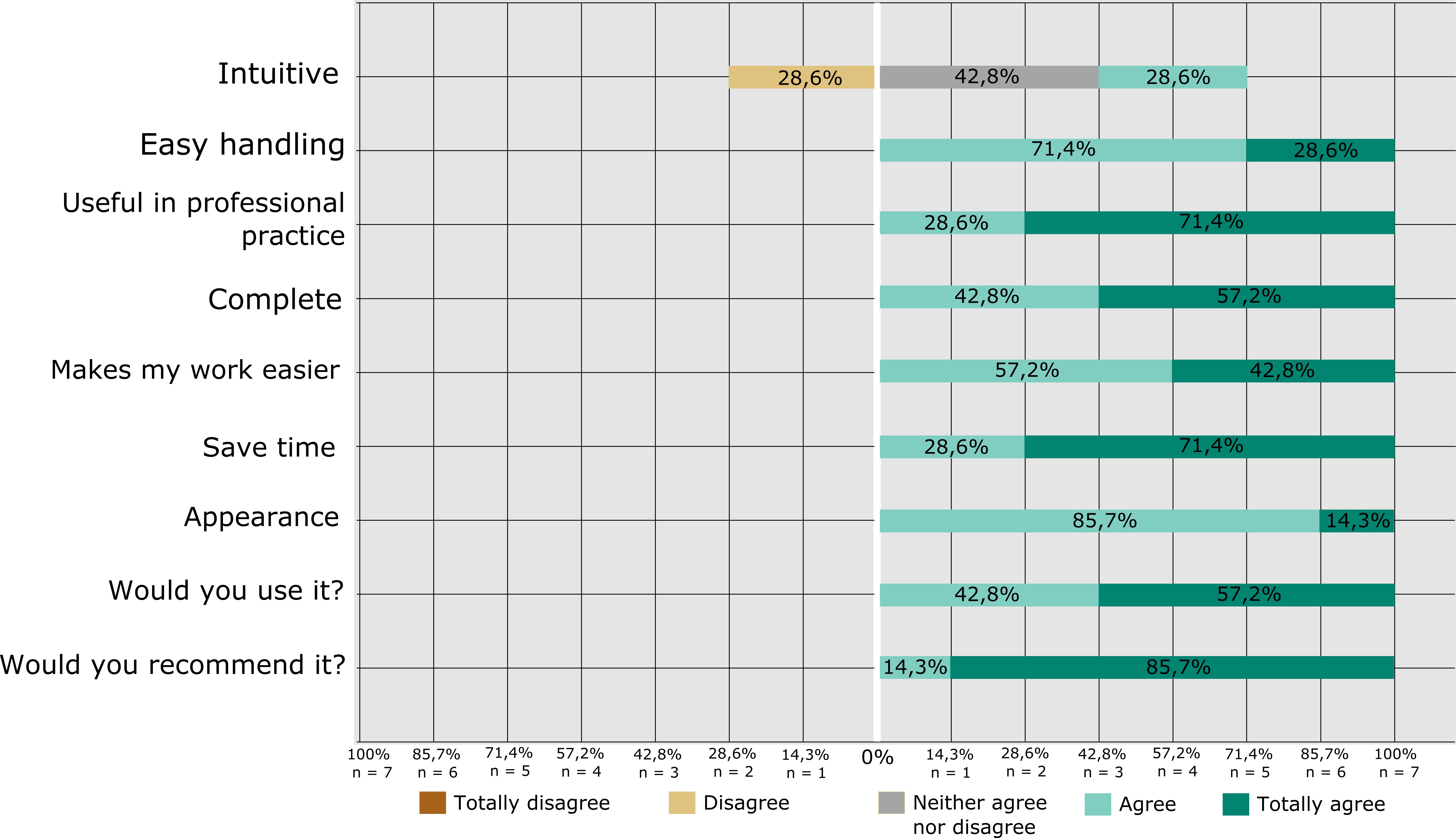

Supplement: Supplementary Figure 1 — Quantitative experts' assessment on the MAR24 tool. [file Image_1.JPEG]

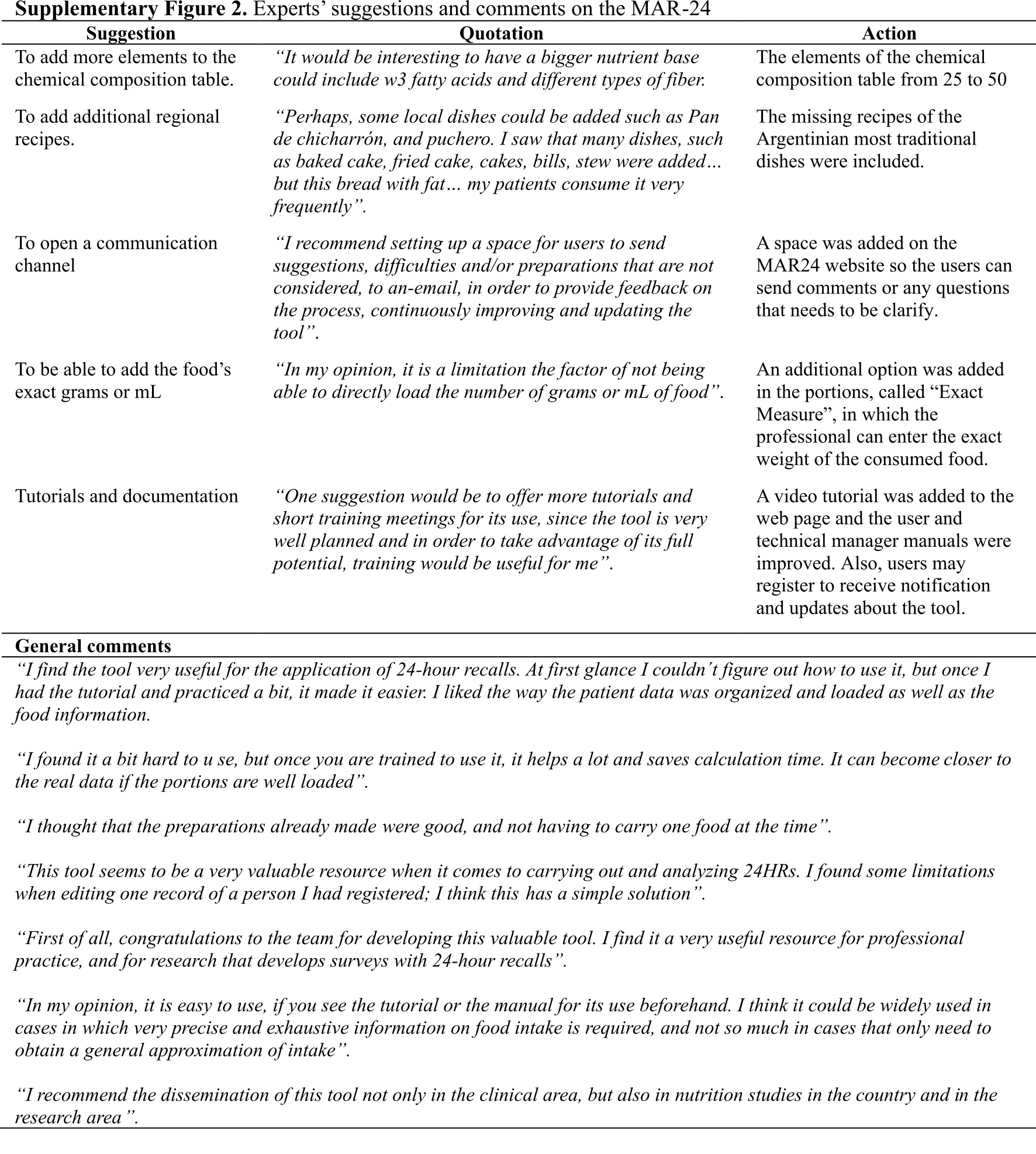

Supplement: Supplementary Figure 2 — Qualitative experts' assessment on the MAR24 tool. [file Image_2.jpg]
